# Intraprocedural contrast-enhanced ultrasound (CEUS) in liver percutaneous radiofrequency ablation: clinical impact and health technology assessment

**DOI:** 10.1007/s13244-014-0315-7

**Published:** 2014-02-22

**Authors:** Giovanni Mauri, Emanuele Porazzi, Luca Cova, Umberto Restelli, Tania Tondolo, Marzia Bonfanti, Anna Cerri, Tiziana Ierace, Davide Croce, Luigi Solbiati

**Affiliations:** 1 IRCCS Policlinico San Donato, Unit of Radiology, Piazza Malan 2—20097 San Donato Milanese, Milano, Italy; 2 CREMS (Centre for Research on Health Economics, Social and Health Care Management), University Carlo Cattaneo—LIUC, Castellanza, VA Italy; 3 Azienda Ospedaliera Ospedale di Circolo di Busto Arsizio, Department of Oncology, Unit of Interventional Oncologic Radiology, Busto Arsizio, Varese Italy; 4 School of Public Health, Faculty of Health Sciences, University of the Witwatersrand, Johannesburg, South Africa

**Keywords:** Radiofrequency ablation, Hepatocellular carcinoma, Cost-effectiveness, Clinical impact, Budget impact, Health technology assessment, Intraprocedural contrast-enhanced ultrasound

## Abstract

**Objectives:**

To assess the clinical and the economic impacts of intraprocedural use of contrast-enhanced ultrasound (CEUS) in patients undergoing percutaneous radiofrequency ablation for small (<2.5 cm) hepatocellular carcinomas.

**Methods:**

One hundred and forty-eight hepatocellular carcinomas in 93 patients were treated by percutaneous radiofrequency ablation and immediate assessment by intraprocedural CEUS. Clinical impact, cost effectiveness, and budget, organisational and equity impacts were evaluated and compared with standard treatment without intraprocedural CEUS using the health technology assessment approach.

**Results:**

Intraprocedural CEUS detected incomplete ablation in 34/93 (36.5 %) patients, who underwent additional treatment during the same session. At 24-h, complete ablation was found in 88/93 (94.6 %) patients. Thus, a second session of treatment was spared in 29/93 (31.1 %) patients. Cost-effectiveness analysis revealed an advantage for the use of intraprocedural CEUS in comparison with standard treatment (4,639 vs 6,592) with a 21.9 % reduction of the costs to treat the whole sample. Cost per patient for complete treatment was € 4,609 versus € 5,872 respectively. The introduction of intraprocedural CEUS resulted in a low organisational impact, and in a positive impact on equity

**Conclusions:**

Intraprocedural use of CEUS has a relevant clinical impact, reducing the number of re-treatments and the related costs per patient.

**Teaching Points:**

• *CEUS allows to immediately asses the result of ablation*.

• *Intraprocedural CEUS decreases the number of second ablative sessions*.

• *Intraprocedural CEUS may reduce cost per patient for complete treatment*.

• *Use of intraprocedural CEUS may reduce hospital budget*.

• *Its introduction has low organisational impact*, *and relevant impact on equity*.

## Introduction

Radiofrequency ablation (RFA) is widely accepted as a treatment for small hepatocellular carcinomas (HCC), with efficacy comparable to surgical resection for these lesions [1–6]. However, complete coagulation necrosis of the index tumour with a sufficient ablative margin is necessary before an RFA treatment can be considered complete [7, 8]. If only partial necrosis is achieved, future re-treatment may be necessary, which may result in increased patient discomfort, greater technical difficulties, higher failure rates and an increased possibility of complications.

Lesion targeting and intraprocedural monitoring are generally performed using non-contrast-enhanced imaging, including ultrasound (US), computed tomography (CT) and magnetic resonance imaging (MRI). On the other hand, precise assessment of technical success requires the use of contrast-enhanced imaging, and is generally performed using contrast-enhanced CT (CE-CT) and/or contrast-enhanced MRI (CE-MRI) within 1 week after the procedure [4, 9–12]. The use of contrast-enhanced US (CEUS) to assess the results of liver RFA has been described, and a diagnostic accuracy similar to CE-CT has been reported [13–18]. For this reason CEUS immediately after ablation has been proposed as an appropriate technique to identify possible incomplete ablation and the need for re-treatment within the same operative session [18, 19]. This approach could theoretically reduce the number of re-treatments required subsequently, with a potentially significant impact on both costs and patient outcomes.

At our institution, CEUS is always performed intraprocedurally immediately following ablation procedures and, when incomplete ablation is depicted, CEUS-guided targeted re-treatment is performed during the same treatment session. The aim of this study was to assess both the clinical and economic impact of intraprocedural CEUS in patients undergoing percutaneous RFA for HCC.

## Materials and methods

### Patients

A retrospective review was performed of consecutive patients that had undergone percutaneous thermal ablation of liver tumours at our institution between January 2008 and June 2011. Approval was obtained by the Institutional Review Board and patient consent was waived.

All patients with HCC lesions smaller than 2.5 cm in size treated by RFA were included in the analysis. At our institution, in all the patients with HCC smaller than 2.5 cm treatment is planned with a single insertion of the electrode. Patients were excluded if they had undergone treatment for metastatic tumours or had undergone prior treatment by microwave ablation. In addition, patients were excluded if their tumours were undetectable by US or if their tumours were larger than 2.5 cm.

### Procedure

Radiofrequency ablation with US guidance was performed under general anaesthesia, using a 3.5-MHz probe with an incorporated guide and a 17-gauge cooled-tip electrode (Cool-Tip; Valleylab, Burlington, MA) with a 3-cm exposed portion. All procedures were performed by two of three interventional radiologists with more than 10 years’ experience in percutaneous thermal ablations and use of CEUS in the diagnostic and interventional field. CEUS was performed using 2.4 ml sulphur hexafluoride microbubbles (SonoVue, Bracco, Italy) before and immediately after each RFA procedure to monitor and assess the therapeutic result before terminating the treatment session. For all examinations, contrast-specific software (Coherent Contrast Imaging [CCI] and Contrast Pulse Sequencing [CPS], Siemens Acuson, USA; ECI, Siemens, Germany; Contrast Tuned Imaging [CnTI], Esaote, Italy) in continuous mode with very low mechanical index (0.01-0.1) was employed.

Pre-treatment CEUS was performed as an initial step in the RFA session in order to reproduce lesion mapping on CE-CT and CE-MRI, and to allow real-time lesion targeting. Images and/or movie clips were digitally stored to be compared with the immediate post-ablation study. Immediate post-ablation evaluation using CEUS was performed 5–10 min after the assumed completion of the RFA session, with the patient still under general anaesthesia.

If residual enhancement was found in the ablated mass and/or the volume of the devascularised area was considered too small to cover the entire tumour with a sufficiently thick safety margin, the treatment was considered incomplete and a new ablation was performed immediately during the same treatment session, as previously described [19]. CEUS was performed again after the new ablation to confirm the completeness of the treatment. CE-CT or CE-MRI was performed at 24 h in all cases and was used as the reference standard for assessment of technical success.

**Fig. 1:**
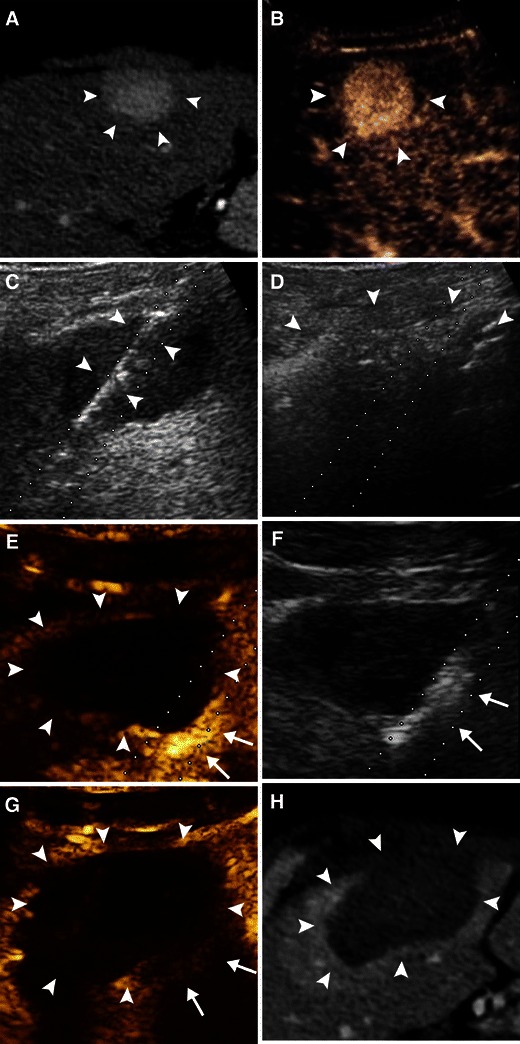
The case of a single HCC that had undergone RFA and immediate re-treatment because of the detection of residual unablated tumour with intraprocedural CEUS. In the left hepatic lobe pre-treatment CE-CT (**a**) and CEUS (**b**) show an HCC with typical hypervascularity in arterial phase (*arrowheads*). **c** The HCC is treated with single insertion of RF electrode (*arrowheads*). **d** Gas produced by heating during ablation (*arrowheads*) seems to diffuse beyond the tumour margins. **e** Intraprocedural CEUS performed few minutes after electrode withdrawal demonstrates residual enhancing viable tumour (*arrows*) at the periphery of the volume of necrosis (*arrowheads*). **f** Second insertion of RF electrode is performed aiming at the area of residual enhancement (*arrows*) (*dotted line* path of the electrode). **g** Post-ablation CEUS demonstrates large volume of necrosis (*arrowheads*) with complete ablation of the residual tumour previously detected (*arrows*). **h** Twenty-four-hour post-ablation CE-CT confirms that treatment is complete (*arrowheads* ablated zone)

A case with positive findings of intraoperative CEUS that modified the treatment is shown in Fig. 1

### Clinical impact

The number of patients in whom intraprocedural CEUS revealed incomplete treatment at the end of the ablation and who underwent a second CEUS-guided ablation in the same interventional session was retrospectively evaluated on the basis of the description of the procedure recorded in the operative registry. The number of incompletely treated lesions was determined by the 24-h post-ablation CE-CT or CE-MRI, read by the same three radiologists with more than ten years’ experience. Patients in whom CEUS revealed incomplete ablation and who underwent a second CEUS-guided ablation in the same session with complete necrosis at 24-h follow-up were considered to have avoided a second intervention as a result of the intraprocedural use of CEUS.

### Health technology assessment

The economic and managerial analysis was conducted using a health technology assessment approach, based on the Guidelines for the Economic Evaluation of Health Technologies [20]. A cost-effectiveness analysis was conducted, along with a budget impact analysis, and analyses of the impacts on the organisation and on equity. The effectiveness parameter taken into account for the cost-effectiveness analysis was the percentage of patients who avoided a second session of treatment due to incomplete ablation.

The costs associated with the procedures were collected with a bottom-up approach, using Activity Based Costing [21–23]. Each procedure was divided into single phases and the related costs of each phase were evaluated, considering direct and indirect costs referring to human resources, machines (8-year amortisation), surgical instruments, consumption materials, drugs and overheads. All phases, from pre-admission to patient discharge for a standard patient without complications, were evaluated using retrospective data related to the study sample in terms of time needed for percutaneous ablation, considering the number of lesions and length of hospitalisation. To assess procedure-related costs without intraprocedural CEUS, a differential analysis was performed, removing time and resources necessary to perform CEUS.

The perspective taken into consideration was that of the hospital. Cost data, provided by the management control service of the hospital, are value added tax inclusive and refer to 2011.

A sensitivity analysis was conducted to test the robustness of the results. One thousand simulations were performed, varying each cost category and effectiveness data with uniform distributions as follows: human resources ±10 %; machinery 0, +750 % (most of the technologies used for the intervention analysed completed the amortisation period, therefore a high range for sensitivity analysis was considered); laboratory exams ±5 %; consumables ±10 %; drugs ±5 %; hotel services ±5 %; overheads 0, -5 %; effectiveness ±5 %. Cost variations were the same for the two procedures in each simulation, while effectiveness could have different values in the same simulation.

The impact on the hospital budget was calculated considering the annual number of RFA procedures for HCC performed in 2010. Two scenarios were than taken into consideration:

- Assessment of the impact of the use of CEUS, using the real number of interventions and reinterventions due to incomplete ablations within the period.
- Assessment of the impact of the procedure without intraprocedural CEUS, considering the treatments and the hypothetical number of re-treatments resulting from incomplete ablations.

The costs of each ablation and re-treatment were calculated for both procedures, along with the diagnosis-related group (DRG) reimbursement provided by the Regional Healthcare Service of Lombardy Region.

A sensitivity analysis was performed using bootstrapping methodology to simulate 100 different samples, considering a uniform distribution of ±10 % in the number of patients with one and two lesions.

The impact on the organisation was assessed through guided interviews with three experienced radiologists of the Interventional Oncologic Department of the hospital. It allowed quantification of the economic impact of the use of intraprocedural CEUS on the organisation and the perception of the short-term and medium/long-term organisational impact, using a seven-point scale (from highly negative to highly positive), compared with ablations without intraprocedural CEUS. The parameters taken into consideration were related to human resources, space needed, software or hardware needs, etc.

The impact on equity was investigated through guided interviews with the three experienced radiologists, detecting their perception using a seven-point scale concerning the two different processes in terms of adverse events, patient safety, accessibility, waiting lists, usability and invasiveness.

## Results

During the course of the study, 384 patients underwent percutaneous RFA for 640 liver lesions. Of these, 148 HCC lesions in 93 patients met the inclusion criteria and entered our analysis. Among the 93 patients, 59 (63.4 %) were men and 34 (36.6 %) women, with an age range of 72.3 ± 7.9 years (median, 73.5 years). Treated HCC lesions had a maximum diameter of 1.86 ± 0.57 cm (median, 2.0 cm). A single HCC was treated in 58 (62.4 %) patients, while two, three and four HCC lesions were treated in 23 (24.7 %), 8 (8.6 %) and 4 (4.3 %) patients, respectively.

### Clinical impact

Intraprocedural CEUS detected incomplete treatment after the first ablation in 43/148 (29.0 %) lesions in 34/93 (36.5 %) patients. All 34 patients underwent additional treatment during the same session until complete absence of vascularisation was demonstrated by intraprocedural CEUS. At 24-h CE-CT or CE-MRI, complete ablation was found in 143/148 (96.6 %) lesions in 88/93 (94.6 %) patients. Only 5/93 (5.4 %) patients subsequently underwent local re-treatment. Thus, as a result of intraprocedural CEUS, a second session of treatment was spared in 29/93 (31.1 %) patients.

### Health technology assessment

The results of the cost-effectiveness analysis are reported in Table 1. The mean cost of RFA without intraprocedural CEUS was € 4,228, which was 3.7 % less than RFA with intraprocedural CEUS. This difference was due to the longer time length of the RFA procedure and to the additional use of contrast agent.

**Table 1:** Cost-Effectiveness Analysis Results

Procedure	Mean cost (€)	Effectiveness	∆ cost (€)	∆ effectiveness	Cost effectiveness
Without intraprocedural CEUS	4,228	64.13 %	4,228	64.13 %	6,592
With intraprocedural CEUS	4,387	94.57 %	+ 159	+ 30.43 %	4,639

The mean cost per procedure is lower without the use of intraprocedural CEUS, while the effectiveness value has a substantial increase with the use of the aforementioned diagnostic procedure. The cost effectiveness value shows a lower, and then favourable, value for the procedure with the use of intraprocedural CEUS

**Fig. 2:**
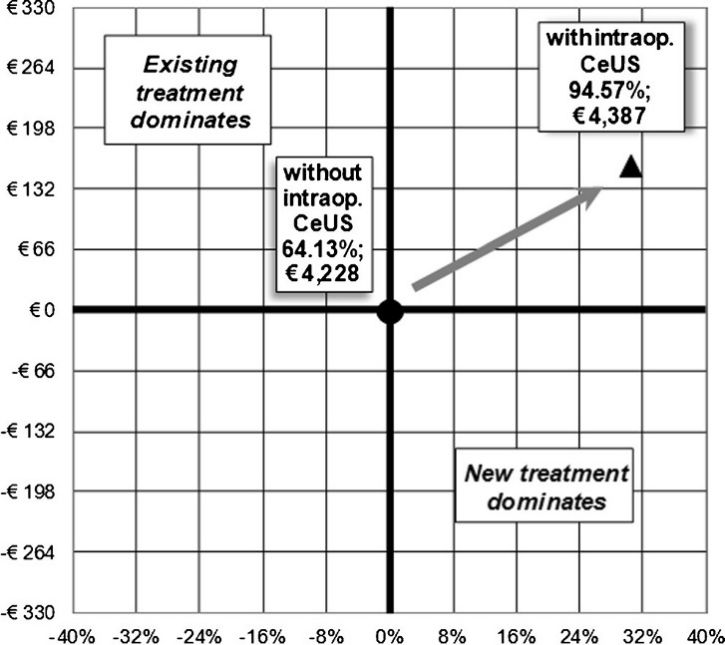
Incremental cost-effectiveness plan. The plan shows the incremental cost and effectiveness of the procedure with intraoperational CEUS, compared with the standard procedure. There is an increase in effectiveness and in costs, the procedure being located in the North-East quadrant. The acceptability of the use of the procedure depends on the willingness to pay of the payer

**Fig. 3:**
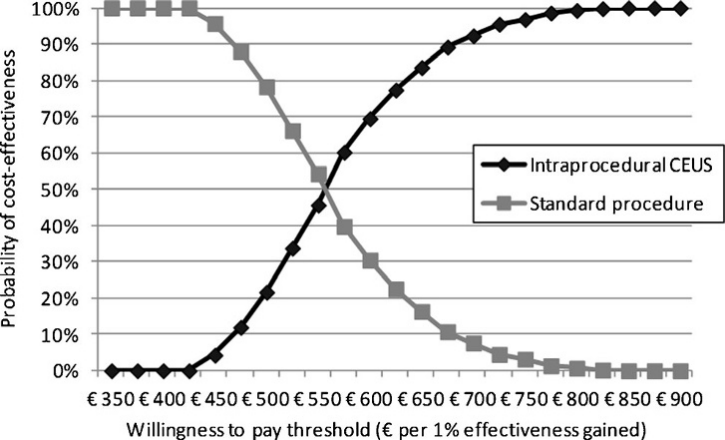
Incremental cost-effectiveness ratio (ICER) sensitivity analysis results. The figure shows the percentage of the 1,000 ICERs calculated with the sensitivity analysis performed, which are cost effective (compared with the other procedure), considering hypothetical willingness to pay values for the regional healthcare service to increase the effectiveness of 1 unit. The cost effectiveness acceptability curve shows a probability higher than 50 % for the procedure with intraprocedural CEUS to be cost-effective, with a willingness to pay per additional effectiveness unit of € 575

Considering the effectiveness of RFA with and without intraprocedural CEUS in terms of avoided reinterventions, the use of intraprocedural CEUS led to a 94.57 % value versus 64.13 % (30.43 % less) when CEUS was not employed. The cost-effectiveness assessment revealed a significant advantage to the use of intraprocedural CEUS (4,639 vs 6,592) (Fig. 2). Though more expensive, the procedure with intraprocedural CEUS proved to be more effective, with an incremental cost-effectiveness ratio (ICER) (that represents the cost per effectiveness point gained) of 521.97 per intervention. The sensitivity analysis results referred to the ICER are reported in Fig. 3. The probability of intraprocedural CEUS being cost-effective was over 50 %, with a willingness to pay a threshold of € 575. This represents the willingness to pay of the Health Service to reach a 1-point effectiveness gain.

The budget impact analysis demonstrated that the use of intraprocedural CEUS allowed a reduction in terms of cost of 21.95 % compared with treatment without intraprocedural CEUS, due to avoided re-treatments. A 25.95 % reduction in terms of reimbursement was also observed, since the number of re-treatments in the sample considered is higher without intraprocedural CEUS and, therefore, the considered treatments and DRGs are higher in this scenario. The mean cost per patient for complete treatment (including first and second RFA) was € 4,609 with the use of intraprocedural CEUS versus € 5,872 for the standard procedure.

The sensitivity analysis revealed minimum and maximum cost reductions for the hospital of 19.02 % and 23.26 %, respectively, with the use of intraprocedural CEUS.

**Fig. 4:**
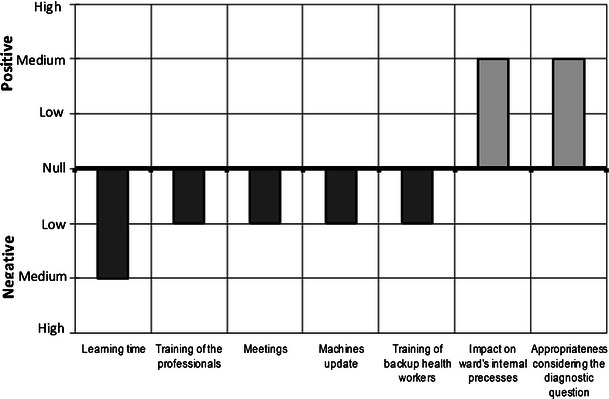
Perceived short-term organisational impact. The use of intraprocedural CEUS leads to a short-term medium negative impact on learning time, and a low negative impact on training for personnel directly involved in the procedure, support personnel, meetings within the department and software update. It leads to a positive medium impact on the internal processes of the ward and appropriateness of requests for diagnostic exams, leading to a reduction in terms of further interventions or investigations needed for the same patient

The analysis of the organisational impact led to the identification of hypothetic investments necessary for the introduction of CEUS within an interventional radiology department of less than € 11,000. Investments were considered to mainly involve software update, training of radiologists and meetings. The perceived short-term organisational impact results are reported in Fig. 4. In the short term, the introduction of CEUS leads to a medium negative impact on learning time, a low negative impact on training for personnel directly involved in the procedure, and for support personnel, meetings within the department (leading to an hypothetical loss of productivity for all the staff involved) and software update. A positive medium impact was determined for internal processes and appropriateness of requests for diagnostic exams, both leading to a reduction in terms of further interventions or investigations needed for the same patient. Considering a medium/long-term perspective, the positive impacts remain while the negative impacts do not further affect the organisation.

Considering the impact on equity, the use of intraprocedural CEUS was associated with a highly positive impact for the quality of data related to the investigated diagnostic question, which leads to more precise information to support medical decisions, a medium positive impact on reductions in waiting lists for surgical interventions, avoiding re-treatments, and a low negative impact on usability and invasiveness of the procedure.

## Discussion

Our results show that the intraprocedural use of CEUS can reduce the number of incomplete treatments and consequently the number of re-treatments, as well as the total costs of RFA for HCC. RFA has been shown to be effective in the treatment of small HCC lesions, with results comparable to surgical resection [1–6]. However, given that the assessment of the completeness of ablation is generally performed after the treatment, often more than one ablation session is required to achieve complete response [9, 10], with increasing risk of complications for the patient and increasing costs. In our series we achieved complete ablation of 96.6 % of lesions in 94.6 % of patients at 24 h, which is comparable with results reported in the literature [1–5].

CEUS can be performed in the operating room after the ablation, and has been used in different fields for the immediate assessment of completeness of ablation [19, 24]. Particularly, CEUS has been proven to be as accurate as other contrast-enhanced imaging modalities for the assessment of technical success after RFA [13–18]. For this reason some authors recommend CEUS immediately after RFA to permit re-treatment during the same interventional session if incomplete ablation is detected [17–19]. We adopted this strategy at our institution and, by using CEUS immediately after RFA, were able to detect incomplete ablation in 29.0 % of lesions in 36.5 % of patients. In these patients we were able to perform an immediate CEUS-guided targeted re-treatment that led to complete ablation in 94.6 % of patients based on 24-h follow-up. Thus, a second session of treatment was avoided in 31.1 % of patients. The need of a second intervention—that we performed in the same session—in our group is slightly higher than other series reported in the literature [1–9]. Several factors may have contributed to this result. Firstly, being a second-level centre, with high expertise in the treatment of liver diseases, patients are often referred to our department from other hospitals. Thus, our group included a high proportion of “complex cases” compared with cases at more general interventional radiology departments, with an increased mean technical difficulty of cases to be treated. Moreover, 37.6 % of patients in our series were treated for more than one tumour in the same session. Third, the result of CEUS was judged by the interventional radiologist performing the treatment, and equivocal cases were generally considered as incompletely ablated, and underwent a second ablative treatment. Thus, the rate of incomplete treatment after the first ablation may have been slightly overestimated. Moreover, we finally achieved complete ablation at 24 hours in 94.6 % of patients.

From an economic point of view, compared with the standard procedure, the introduction of intraprocedural CEUS resulted in a 3.7 % increase in costs, due to a longer procedural time and the cost of the contrast agent. However, the use of intraprocedural CEUS resulted in better cost-effectiveness compared with the standard procedure and a higher probability of being cost-effective, with a € 575 threshold of willingness to pay.

From a hospital perspective, the intraprocedural use of CEUS resulted in a 21.95 % reduction in costs due to a lower number of re-treatments. This would result in the possibility of performing ablations on a greater number of patients, with consequently more effective use of resources and shortening of waiting lists. From the point of view of the Health Service, the use of intraprocedural CEUS allows a lower cost per patient for complete treatment, resulting in savings for the entire service.

The introduction of intraprocedural CEUS in centres where CEUS is already employed for other purposes would result in a low organisational impact, but to a relevant impact on equity, since the avoided re-treatments would have a positive impact on the quality-of-life of patients, decreasing their stress levels and reducing the number of hospitalisations.

Even though CEUS has a diagnostic accuracy similar to CE-CT in the assessment of treatment results [13–18], there are only few studies that compare CEUS with other contrast-enhanced modalities immediately after RFA. A second possible limitation of our study is that being a second-level centre, with a larger number of “complex cases” compared with more general interventional radiology departments, the same analysis performed in a different hospital might lead to different results. Moreover, the decision to perform a new ablation immediately, in the same session, was made on the basis of the critical analysis of the result of intraprocedural CEUS made by the interventional radiologists. In some cases with an easy path to the target and a safely located tumour, even with equivocal result of incomplete ablation at CEUS a second ablation was performed. Thus, the need for immediate re-treatment and, consequently, the clinical and economic impacts of CEUS, could have been overestimated in our series. As a consequence, unnecessary repetition of treatment in a significant number of patients would have happened. A fourth limitation is the absence of a true control group in which CEUS was not performed, as our results were obtained by comparing a real group in which CEUS was performed with a hypothetical one in which CEUS was not performed. A further bias in our study derives from the inclusion of only small (<2.5 cm) HCCs in our series.

In conclusion, the intraprocedural use of CEUS may have a relevant clinical impact in reducing the number of second ablative sessions needed to achieve a complete ablation of the tumour to be treated. Moreover, the intraprocedural use of CEUS may significantly reduce the costs per patient related to the percutaneous treatment with RFA of patients with HCC, being also characterised by a low organisational impact, and in a relevant impact on equity
